# Mitochondrial genomes reveal an explosive radiation of extinct and extant bears near the Miocene-Pliocene boundary

**DOI:** 10.1186/1471-2148-8-220

**Published:** 2008-07-28

**Authors:** Johannes Krause, Tina Unger, Aline Noçon, Anna-Sapfo Malaspinas, Sergios-Orestis Kolokotronis, Mathias Stiller, Leopoldo Soibelzon, Helen Spriggs, Paul H Dear, Adrian W Briggs, Sarah CE Bray, Stephen J O'Brien, Gernot Rabeder, Paul Matheus, Alan Cooper, Montgomery Slatkin, Svante Pääbo, Michael Hofreiter

**Affiliations:** 1 Max Planck Institute for Evolutionary Anthropology, Deutscher Platz 6, D-04103 Leipzig, Germany; 2 Department of Integrative Biology, University of California, Berkeley, CA 94720-3140, USA; 3 Department of Ecology, Evolution and Environmental Biology, Columbia University, 1200 Amsterdam Avenue, MC5557, New York, NY 10027, USA; 4 Sackler Institute for Comparative Genomics, American Museum of Natural History, Central Park West at 79th Street, New York, NY 10024, USA; 5 Departamento Científico Paleontologia de Vertebrados, Museo de La Plata. Paseo del Bosque, (1900) La Plata, Buenos Aires, Argentina; 6 MRC Laboratory of Molecular Biology, Hills Road, Cambridge, CB2 2QH, UK; 7 Australian Centre for Ancient DNA, School of Earth and Environmental Sciences, University of Adelaide, Adelaide, SA 5005, Australia; 8 Laboratory of Genomic Diversity, National Cancer Institute, Frederick, MD 21702-1201, USA; 9 Department of Paleontology, University of Vienna, 1090 Vienna, Austria; 10 Alaska Quaternary Center, University of Alaska Fairbanks, Fairbanks, AK 99775, USA

## Abstract

**Background:**

Despite being one of the most studied families within the Carnivora, the phylogenetic relationships among the members of the bear family (Ursidae) have long remained unclear. Widely divergent topologies have been suggested based on various data sets and methods.

**Results:**

We present a fully resolved phylogeny for ursids based on ten complete mitochondrial genome sequences from all eight living and two recently extinct bear species, the European cave bear (*Ursus spelaeus*) and the American giant short-faced bear (*Arctodus simus*). The mitogenomic data yield a well-resolved topology for ursids, with the sloth bear at the basal position within the genus *Ursus*. The sun bear is the sister taxon to both the American and Asian black bears, and this clade is the sister clade of cave bear, brown bear and polar bear confirming a recent study on bear mitochondrial genomes.

**Conclusion:**

Sequences from extinct bears represent the third and fourth Pleistocene species for which complete mitochondrial genomes have been sequenced. Moreover, the cave bear specimen demonstrates that mitogenomic studies can be applied to Pleistocene fossils that have not been preserved in permafrost, and therefore have a broad application within ancient DNA research. Molecular dating of the mtDNA divergence times suggests a rapid radiation of bears in both the Old and New Worlds around 5 million years ago, at the Miocene-Pliocene boundary. This coincides with major global changes, such as the Messinian crisis and the first opening of the Bering Strait, and suggests a global influence of such events on species radiations.

## Background

The bear family (Ursidae) is one of the most studied families within the order Carnivora. Members of this family are present on most continents and occupy a wide range of ecological niches from the arctic ice shelves to tropical rainforests (see Additional File 1, Figure S1a). Despite numerous morphological and molecular studies on the phylogenetic relationship among Ursidae members, no consensus exists with regard to either their phylogeny or their taxonomic nomenclature (Table 1). Most analyses have concentrated on the eight extant bear species: brown bear, American black bear, Asian black bear, polar bear, sun bear, sloth bear, spectacled bear and giant panda (for species names see Table 1). Molecular studies based on mitochondrial and nuclear DNA from modern bears have recently provided convincing evidence about several of the controversial relationships among the bears, such as the basal positions of the giant panda and the spectacled bear in the bear tree [1-4] and the position of the polar bear within the brown bear tree making the later paraphyletic [5,6]. However, molecular studies for a long time failed to conclusively resolve the phylogenetic relationships among the members of the bear subfamily *Ursinae *[5], which includes all living bear species except the giant panda and the spectacled bear, from here on referred to as ursine bears. The phylogenetic uncertainty has resulted in major taxonomic confusion. Based on both morphological and molecular data up to six different genera (*Ursus*, *Helarctos, Euarctos, Selenartos, Thalarctos *and *Melursus*; i.e. one for each species) have been suggested for the extant ursine bears (Table 1).

**Table 1:** Taxonomic designations for the bears.

Common name	Eisenberg [72]	Ewer [73]; Corbet & Hill [74]	Zhang & Ryder [75]	Thenius [76]; Wozencraft [77]	Hall [28]; Nowak [29]; Yu [4], this study
giant panda	*Ailuropoda melanoleuca*	*A. melanoleuca*	*A. melanoleuca*	*A. melanoleuca*	*A. melanoleuca*
spectacled bear	*Tremarctos ornatus*	*T. ornatus*	*T. ornatus*	*T. ornatus*	*T. ornatus*
Asian black bear	*Selenarctos thibethanus*	*S. thibethanus*	*S. thibethanus*	*Ursus thibethanus*	*U. thibethanus*
sloth bear	*Melursus ursinus*	*M. ursinus*	*M. ursinus*	*M. ursinus*	*Ursus ursinus*
sun bear	*Helarctos malayanus*	*H. malayanus*	*H. malayanus*	*H. malayanus*	*Ursus malayanus*
polar bear	*Thalarctos maritimus*	*T. maritimus*	*Ursus maritimus*	*U. maritimus*	*U. maritimus*
American black bear	*Ursus americanus*	*Euarctos americanus*	*E. americanus*	*U. americanus*	*U.americanus*
brown bear	*Ursus arctos*	*U. arctos*	*U. arctos*	*U. arctos*	*U. arctos*

Recently, a study on mitochondrial genome sequences (mtDNAs) of all extant bears presented for the first time an almost completely resolved bear phylogeny with support for most of the problematic nodes in the bear family tree, except for the position of the sloth bear [4]. This shows that longer sequences are necessary for reconstructing a robust phylogeny [4,6-9]. Such large data sets also facilitate the molecular dating of divergence events within a phylogeny [12-14]. To resolve the relationships between the extant and extinct members of the bear family and to date the various divergence events among them, we used the complete mtDNA (consisting of ~17 kb) from ten different bear species. In addition to three published modern mtDNAs [10], we amplified and sequenced five modern bear mtDNAs using a 2-step multiplex PCR approach [9,16]. We also amplified and sequenced entire mtDNAs from the extinct European cave bear (*Ursus spelaeus*), believed to belong to the ursine bears [11], and the extinct North American giant short-faced bear (*Arctodus simus*) (see Additional File 1, Figure S1b), thought to be related to the spectacled bear [12].

## Results

### Sequence retrieval

We retrieved complete mtDNAs from GenBank for three extant bear species: brown bear, American black bear and polar bear (GenBank: NC003427, GenBank: NC003428, GenBank: NC003426). For the remaining five living bear species, we sequenced the entire mtDNA in overlapping fragments using a 2-step multiplex PCR approach [7] and a mixture of direct sequencing and sequencing multiple clones (EMBL:FM177759, EMBL:FM177761, EMBL:FM177763, EMBL:FM177764, EMBL:FM177765). We also obtained the complete mtDNA from the extinct European cave bear using a 44,000 year old bone found in Gamssulzen Cave, Austria. Again, we used a 2-step multiplex approach, but in this case, all PCR products were cloned and multiple clones were sequenced (EMBL:FM177760). Moreover, to ensure sequence accuracy, we determined each sequence position from at least two independent primary PCRs [13]. When we observed a discrepancy between the consensus sequences from each of the two amplifications we performed a third amplification and used the consensus sequence from all three amplifications (see Additional File 1). We used the same approach to sequence the extinct American giant short-faced bear mtDNA, using a 22,000 year-old calcaneum bone from Eldorado Creek, Canada (EMBL:FM177762). In order to further ascertain that the results obtained are reproducible, samples of both extinct bears were extracted, amplified and sequenced each in an additional laboratory that did not have access to the results obtained in Leipzig. For the cave bear a total of 3,520 bp were independently reproduced in Cambridge and for the American giant short-faced bear a total of 395 bp was replicated in the Australian Centre for Ancient DNA in Adelaide. The consensus sequences for all fragments determined in Cambridge were identical to those determined in Leipzig. The replicated fragments at the Australian Centre for Ancient DNA were identical to the sequence obtained in Leipzig except for a single deletion close to the 5'-end of the light strand in the first fragment. The sequence for this fragment was obtained by direct sequencing in just one 5' to 3' direction on the light strand. Given that sequence accuracy immediately downstream the sequencing primer is low, it is likely that this deletion represents a sequencing artifact.

### Phylogenetic analyses

**Figure 1:**
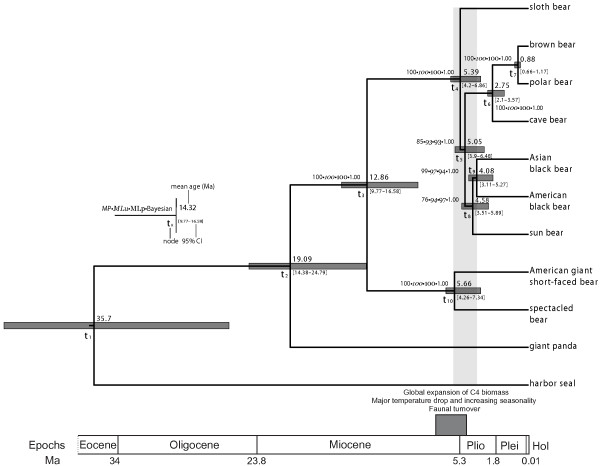
**Maximum clade probability tree displayed as a chronogram from the BEAST analysis of the unpartitioned mitochondrial genome alignment**. All lineages evolved according to a strict clock and the GTR+Γ_4 _substitution model. Numbers above the nodes indicate phylogenetic support measures. Node bars illustrate the width of the 95% highest posterior density. Numbers in bold indicate the posterior mean estimates of divergence times.

All ten bear mtDNAs were aligned using the harbor seal (*Phoca vitulina*) mtDNA as outgroup. Phylogenetic trees were reconstructed using maximum parsimony (MP), maximum likelihood (ML) and Bayesian inference. We recovered the same topology using all above-mentioned optimality criteria (Figure 1 and Table 2). Our results confirm the giant panda's basal position in the bear phylogeny [1-4,14-17] and also place the spectacled bear outside ursine bears. In contrast to previous studies our data was sufficient to resolve the phylogenetic relationships among ursine bears with statistical support for all nodes. The sloth bear falls basal to all other ursine bears, which form a monophyletic group with 85%, 93%, and 100% support (MP symmetric resampling, ML bootstrap, and 1.00 Bayesian posterior probability (PP), respectively). The hypothesis suggesting that the sloth bear is basal to the sun bear and black bear clade (hypothesis 6, see Additional File 1, Figure S2) did not have a significantly worse likelihood (AU test, *p *= 0.147) than the topology favored by our data (sloth bear as the most basal ursine bear; hypothesis 9, see Additional File 1, Figure S2) although it had a slightly higher homoplasy index and a less parsimonious tree (HI_best _= 0.389, HI_competing _= 0.393; TL_best _= 10092, TL_competing _= 10157). In a MP analysis, however, this hypothesis (hypothesis 6, see Additional File 1, Figure S2) received significantly less support (Wilcoxon signed-ranks and sign tests, *p *< 10^-4^). Thus, we suggest that ursine bears are separated into two sister clades, comprised of three species each with the sloth bear forming the basal branch. The first clade contains cave, brown, and polar bears and is supported by all methods. The second clade is composed of the sun and black bears (American and Asian) and receives varying support values, depending on the tree reconstruction method (76% MP symmetric resampling, 97% ML bootstrap, and 1.00 Bayesian PP). Finally, the placement of the extinct American giant short-faced bear as a sister taxon to the spectacled bear is supported in all analyses (100% bootstrap/symmetric resampling and 1.00 PP in all analyses).

**Table 2:** Node support values for the mitogenomic phylogeny of the bears.

Node	Unpartitioned			Partitioned	
		
	MP	ML	Bayesian	ML	Bayesian
	
t_3_	100	100	1.00	100	1.00
t_4_	100	100	1.00	100	1.00
t_5_	85	93	1.00	93	1.00
t_6_	100	100	1.00	100	1.00
t_7_	100	100	1.00	100	1.00
t_8_	76	94	1.00	97	1.00
t_9_	99	97	1.00	94	1.00
t_10_	100	100	1.00	100	1.00

Unpartitioned and partitioned phylogenetic analyses in maximum parsimony (MP), maximum likelihood (ML) and Bayesian inference of phylogeny. In partitioned analyses every partition was allowed to evolve under a separate unlinked GTR+Γ substitution model and distinct base frequencies in ML and Bayesian inference of phylogeny.

**Figure 2:**
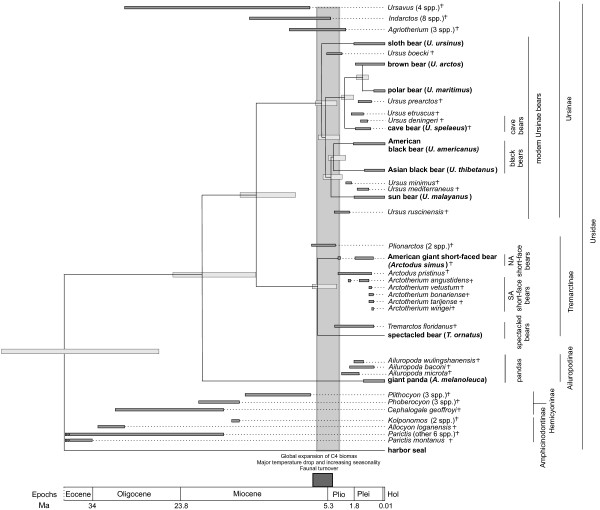
**Temporal ranges of extant and fossil bears**. Extinct genera and species are denoted with a cross (†). Species used in this study are written in bold. Horizontal dark grey bars indicate temporal range based on fossil evidence. Horizontal light grey bars show width of the 95% credibility interval for the molecular divergence time (see also Figure 1). The vertical grey bar illustrates the range of the posterior mean estimate of divergence times for all extant ursine bears (expect polar and brown bear) as well as American giant short-faced bear and spectacled bear. The dark grey box illustrates a time interval of massive global changes around the Miocene- Pliocene boundary.

The phylogenetic reconstruction makes evident a difficulty in resolving the relationships within ursine bears. As a matter of fact, most of the internal branches are very short. This observation (Fig. 2) makes it likely that individual genes (or short sequences) may exhibit different tree topologies, as shown for humans, chimpanzees and gorillas [18]. In our case, we notice that individual loci of the mtDNA support different topologies. Genes such as 12S rRNA, ND4L, ND5, and ND3 exhibited phylogenetic incongruence with two to four other mitochondrial genes (ILD test, *p *≤ 0.05). The highest amount of phylogenetic conflict emerged from partitioning the mtDNA to individual genes and the tRNAs of the two strands (tRNA^- ^and tRNA^+^), but only two nodes showed evidence of hidden conflict in a MP setting (node t_3 _HBS = -10, node t_5 _HBS = -5). Emerging support was evident for all terminal nodes including the ursine branching point (HBS range: 7–21). Intra-ursine nodes t_5_, t_8_, and t_9 _were the only nodes to show <100% replicate-based support values, a fact that may be owed to the lack of consistent support provided by those genes (PHBS range: -1 to -7).

### Estimation of divergence times

We used several fossil calibration points to estimate the mtDNA divergence dates within the bears (see Materials and Methods). The posterior mean of the divergence time between bears and the harbor seal was estimated at 36 million years ago (Ma), which agrees with previous estimates based on both molecular and paleontological data [12,19]. However, due to the wide uniform priors we used for the calibration points, the confidence interval (26.5–47.4 Ma, Table 3) remains large, depending on the type of analysis. The initial divergence within bears occurred between the giant panda and all remaining bears, estimated to have happened between 17.9 and 22.1 Ma (range of posterior means across analyses). The next divergence was that of the New World spectacled bear group, which separated from the main ursine bear lineage about 12.4 to 15.6 Ma. The posterior mean divergence of the two New World species, the extant spectacled and the extinct American giant short-faced bear, was estimated to have happened between 5.3 and 7 Ma. Within the remaining ursine bears, the estimated divergence times all show overlapping confidence intervals, except for those within the brown bear clade.

**Table 3:** Posterior estimates of divergence times.

Node	Unpartitioned				Partitioned	
		
	BEAST		mcmctree		mcmctree	
			
	Mean	95% HPD	Mean	95% HPD	Mean	95% HPD
	
t_1_	35.69	26.55–46.51	36.59	30.71–42.63	36.49	31.20–47.40
t_2_	19.09	14.38–24.79	22.05	18.37–25.57	17.88	15.13–23.23
t_3_	12.86	9.77–16.58	15.57	12.93–17.99	12.36	10.44–15.97
t_4_	5.39	4.20–6.86	6.31	5.23–7.14	4.88	4.17–6.37
t_5_	5.05	3.90–6.48	5.80	4.81–6.64	4.55	3.85–5.99
t_6_	2.75	2.10–3.57	3.11	2.55–3.63	2.41	2.01–3.23
t_7_	0.88	0.66–1.17	0.97	0.78–1.16	0.75	0.61–1.00
t_8_	4.58	3.51–5.89	5.31	4.39–6.11	4.11	3.44–5.38
t_9_	4.08	3.11–5.27	4.69	3.86–5.43	3.66	3.07–4.84
t_10_	5.66	4.26–7.34	6.98	5.73–8.18	5.33	4.43–7.00
rate	1.199	0.896–1.50	1.07	0.92–1.28	AF	AF
κ	-	-	35.72	33.01–38.63	AF	AF
α	0.155	0.146–0.163	0.20	0.19–0.21	AF	AF

The two mcmctree columns correspond to the unpartitioned and partitioned analyses. The divergence time estimates are in millions of years before present. HPD, highest posterior density; rate, evolutionary rate (× 10^-8 ^substitutions/site/year); κ, transition-transversion parameter of the HKY model; α, shape parameter of the rate heterogeneity Γ-distribution. AF, see Additional File 1.

Within the brown bear clade, we dated the divergence event between the cave and brown bear mtDNA to 2.4–3.1 Ma. The origin of the polar bear is more difficult to determine, as partial mtDNA sequences suggest that polar bears actually fall within the genetic diversity spectrum of brown bears [20,21], where they constitute a monophyletic clade closely related to a clade of brown bears from the ABC Islands in Alaska. Unfortunately, the published brown bear mtDNA [10] does not originate from this ABC Island clade, and therefore our estimated divergence for polar bears and brown bears is not a minimum date for this event, but rather the divergence date for different brown bear clades. As a consequence, the estimated divergence date of 2.7 and 1.3 Ma for brown and polar bears obtained in two recent studies [4,19] using the same complete mtDNAs should be interpreted with care. A mtDNA from an ABC island brown bear will be required to date the actual speciation event of polar bears more accurately.

We also examined whether more sequence data would improve our estimates on divergence times by plotting the posterior means of divergence times against the width of their corresponding 95% credibility interval (see Additional File 1, Figure S3), following Yang and Rannala [22]. We found significant correlations for both the unpartitioned and partitioned datasets (*p *< 2.2 × 10^-16^). This linear relationship strongly suggests that longer sequences or more taxa than those examined here are unlikely to increase the precision of the divergence time estimates. Therefore, in order to narrow the confidence intervals for the divergence date estimates within the bear phylogeny, more precisely dated fossil calibration points would be required.

The estimated substitution rate of approximately 10^-8 ^substitutions/site/year was more similar to a mitogenomic dataset from primates than to the rate from extant and extinct proboscideans [9]. This evolutionary rate was also higher than that for parts of the nuclear IRBP gene in bears (0.139 × 10^-8 ^substitutions/site/year; [17]).

## Discussion

Our study represents the first comprehensive sampling of mtDNAs for recent bears, including all living and two recently extinct bear species. The cave bear and the American giant short-faced bear are the third and fourth Pleistocene species for which mtDNAs have been determined. Moreover, the cave bear genome is the first determined from a Pleistocene sample obtained from a non-permafrost environment. Compared to the extinct moas from which complete mtDNAs have previously been determined from non-permafrost specimens [23], the cave bear genome extends the time frame by an order of magnitude, showing that complete mtDNA analysis can be performed using a wide range of samples. As is common in large scale ancient DNA analyses [7,9,24], we found a number of consistent differences between independent primary PCRs, all of which were either C to T or G to A substitutions (see Additional File 1). This confirms previous reports that deamination of cytosine is one of the most common, and probably the only type of miscoding lesion in ancient DNA [13,24-26]. Moreover, the high number of consistent substitutions (81) observed in the cave bear genome sequences shows that each sequence position needs to be replicated when performing such large scale analyses.

This analysis has allowed the phylogenetic topology of the bear family to be resolved with high support values. Interestingly, it places the sloth bear basal to all other ursine bear species and the sun bear in a sister group related to the two black bear species. The latter observation coincides with paleontological information [27] and previous mtDNA studies [4,17,21]. An earlier study analysing six mtDNA fragments, also placed the sloth bear basal to all other members of the ursine bears [3]. However, this study found weak support for the sun bear as being basal to the brown bear – polar bear clade rather than to the two black bear species.

The phylogenetic reconstruction also reveals the reasons for previous problems in resolving the relationships among ursine bears, as most of the internal branches for their phylogenetic tree are very short. Such a short internal branch structure (Figure 1) makes it likely that individual nuclear genes (or short sequences) may exhibit different tree topologies, as shown for nuclear loci from humans, chimpanzees and gorillas [18]. Furthermore it was previously shown that despite being a non-recombining single genetic locus, individual genes on the mtDNA might produce different tree topologies [6,8,9,18].

The mitogenomic data also has implications for bear taxonomy. Six ursine bears and the sloth bear are monophyletic with absolute support, which agrees with Hall and Nowak's inclusion (Table 1) of the Asian black bear, American black bear, sun bear, polar bear and brown bear within the genus *Ursus *[28,29] and confirms the mitogenomic study by Yu et al [4]. Given the short divergence time of the six ursine bears and the sloth bear we suggest, following Hall 1981, Nowak 1991 and Yu et al 2007 [4,28,29], that the sloth bear is grouped together with the other ursine bears in the genus *Ursus *and that the other genus names previously suggested for members of this radiation are discarded (Table 1).

Using this data set and multiple fossil calibration points, we have dated the various mtDNA divergence events during bear evolution with reasonable confidence. Strikingly, the divergence of the giant panda is estimated at about 19 Ma (95% HPD: 14.4–24.8 Ma, HPD: highest posterior density). This estimate is much earlier than previously reported for the divergence of the panda lineage from the *Ursavus *lineage based on teeth morphology of *Agriarctos *fossils (12–15 Ma) [30]. The latter divergence date has been used in several studies as a calibration point for dating bear radiations [2,4,35]. We decided not to use this date as a calibration point, since the oldest known panda fossil, *Ailuropoda microta*, is less than 2.4 million years old [31], and therefore allows no inference about the date of divergence of this lineage. Moreover, the fossil record for both *Ailuropoda *and its potential ancestral species from the genus *Agriarctos *is sparse, making an early Miocene divergence date for the giant panda's lineage plausible. Interestingly, the next divergence event is not until 13 Ma (spectacled and American giant short-faced bear) followed by a gap until 6 Ma when a rapid radiation occurs. The American giant short-faced and spectacled bears diverged around 5.7 Ma, and the five ursine lineages diverged between 5.4 and 4.1 Ma (posterior mean age estimates) (Figures 1 and 2).

Thus, taking the confidence intervals for the molecular dating into account, seven lineages radiated between 3.7 and 7 Ma. Such rapid radiations are also observed in other mammals, such as the cats [32] and procyonids [33], as well as in bird families like the woodpeckers [34]. Strikingly, the major radiation wave for these families also occurred at the end of the Miocene. In combination with the fossil record, the mtDNA divergence estimates suggest that the rapid radiation of the bear family around the Miocene-Pliocene boundary followed a major extinction of some of the main bear genera such as *Ursavus*, *Indarctos, Agriotherium*, and the *Hemicyoninae *(Figure 2). Similar species turnover events were also observed for other mammals over a limited time span near the Miocene-Pliocene boundary resulting in a massive extinction of more than 60–70% of all Eurasian genera and 70–80% of North American genera [35]. The cause of this widespread species turnover during this time period remains unclear. Some studies suggest that the initial opening of the Bering Strait at the beginning of the Pliocene around 5.3 Ma caused a major separation of northern hemisphere habitats [36]. Major climatic changes occurred during that time, such as the Messinian crisis during which the Mediterranean Sea lost its connection to the world ocean system and became desiccated [37]. These changes resulted in forest cover decline and the spread of arid habitats in Northern America and Eurasia [38,39] as well as a global increase in C4 biomass [40]. During that time, open grassland habitats, which were exploited by an entirely new suite of mammals [40], replaced the earlier less seasonal woodland forest habitats. Thus, it is possible that the environmental changes associated with the Miocene-Pliocene boundary and the following emergence of new ecological niches such as open grasslands caused an adaptive radiation in Old and New World bears similar to a number of other species groups [34]. This could explain the divergence of the *Tremarctinae *with the spectacled bear adapted to closed habitats and the American giant short-faced bears being predators dwelling in open habitats [12,27]. The latter adaptation was also described in other predator species that evolved around the Miocene-Pliocene boundary and were built for hunting in open habitats such as the cats [32,35]. Other events such as the opening of the Bering Strait could have additionally promoted allopatric speciation in black bears. Our divergence time estimates suggest that the American black bear could have spread to America before the Bering Strait opened around 5.3 Ma [36]. An early migration of ursine bears into the Americas is also supported by the oldest known *Ursus *fossil in North America, *Ursus abstrusus *[41], which was dated at 4.3 Ma, suggesting that *U. abstrusus *may be ancestral to the American black bear lineage.

Obviously, the Miocene-Pliocene global changes had a major impact on the radiation of bears and other species, both between and within the Old and New Worlds. It is interesting to note that African apes experienced a similar species turnover at the end of the Miocene, including the divergence of the chimpanzee and human lineages [42]. This latter event has been attributed to a magnified climatic variability starting at the end of the Miocene [43]. More studies are necessary to address the relationships between global changes and species radiations at the beginning of the Pliocene. Our results strongly support the idea of a major wave of bear radiations during that time.

Our data also indicate a much earlier divergence for the cave bear and brown bear lineages than those previously assumed, with a mean estimate at 2.8 Ma. This date agrees with recent results suggesting the existence of representatives of the brown bear lineage in Europe as early as 1.5 Ma (G. Rabeder, personal observation). Nevertheless, it questions other studies suggesting a later divergence time for this species pair at around 1.2–1.4 Ma based on the fossil record [27] and molecular data [44]. Loreille et al. [44], however, used Taberlet & Bouvet's estimated divergence date for the two European brown bear lineages (Western and Eastern) of 850 ka [45], which in turn was based on an application of Vigilant et al.'s [46] intraspecific human rate of 8.4 × 10^-8 ^substitutions/site/year – Taberlet & Bouvet cautioned that their estimates could be prone to uncertainty as they imported a human evolutionary rate. Given recent reports of problems in estimated intraspecific divergence times based on interspecific calibrations and *vice versa*, the implicit use of indirectly extrapolated evolutionary rates is not recommended [51,52].

Most of the youngest fossils for *Ursus etruscus*, the assumed ancestor of the cave and brown bear, have been dated to 2–2.7 Ma [47], suggesting that a late divergence for the two lineages around 1.2 Ma is rather unlikely. These dates also partially overlap with the divergence date we obtained (range of posterior means across methods: 2.4–3.1 Ma). A greater number of reliably dated fossils from early members of both the cave bear and brown bear lineages are necessary to date the divergence of *U. spelaeus*. However, around 2.8 Ma, the climate again changed dramatically with the onset of the first major cooling events and climatic oscillations at the end of the Pliocene that eventually led to the Pleistocene glaciations [48]. Thus, if bear speciation events were influenced by climate change, cave bears and brown bears may indeed have separated as early as 2.8 Ma.

## Conclusion

Using complete mitochondrial genome sequences from both extinct and extant bears, we found evidence for a rapid radiation of bears at the Miocene – Pliocene Boundary 5–6 million years ago within the Old and New worlds. As rapid radiations were also observed in other species groups around this time [37-39], we suggest that climate change played an important role during bear evolution and animal speciation in general.

Our results clearly demonstrate the power of mitogenomic analyses for resolving complicated phylogenetic relationships among both extant and extinct species, using samples obtained not only from permafrost, but also from non-permafrost environments.

## Methods

### Ancient and modern DNA samples

The modern DNA samples of the Asian black bear, sloth bear and sun bear were obtained from DNA stocks held at the National Cancer Institute, Laboratory of Genomic Diversity in Frederick, Maryland (USA). The DNA samples of the giant panda and spectacled bear were obtained from the National Fish & Wildlife Forensic Lab in Ashland, Oregon (USA).

In Leipzig, cave bear DNA was extracted from 640 mg of bone powder taken from a femur found in Gamssulzen cave (Austria) that was dated to 44.160 +1.400/-1.190 BP (KIA 25287). The extraction was performed as described previously [49], yielding 70 μl of DNA extract. In Cambridge, 500 mg of cave bear bone was extracted using the same protocol as in Leipzig. Details for the American giant short-faced bear DNA extraction performed in the Australian Centre for Ancient DNA can be found in Additional File 1.

### Multiplex amplification and sequencing

Primer pairs were designed by aligning the three published mtDNAs of brown bear, polar bear and American black bear [10], and partial mtDNAs from various bear species retrieved from GenBank. The revised Cambridge reference sequence for the human mtDNA [50] was also included in the alignment. For the primers, regions were chosen that are highly conserved among bears, but carry substitutions compared to the modern human sequence, to minimize the risk of human contamination. As previously described for the 2-step multiplex protocol [7,51], the primer pairs for the first and second step were divided into two sets, ODD and EVEN, to avoid amplifying the overlapping fragments between adjacent products. The two primer sets were used in separate 2-step multiplex PCRs, as previously described [7]. The first amplification step was performed in a total volume of 20 μl. Each reaction contained a final concentration of 1x PCR-buffer, 4 mM MgCl_2_, 250 μM of each dNTP, 150 nM of each primer from one set and 2 U *AmpliTaq^® ^*Gold DNA polymerase plus 5 μl of the DNA extract. PCRs were initiated by exposure to 94°C for 9 min, followed by 25 cycles of 20 s at 94°C, 30 s at 52°C and 1 min at 72°C. At the end, a final 4-min extension at 72°C was performed. This amplification was then diluted 40 fold and 5 μl of the dilution were used as a template in each of the single amplification reactions. Reagent concentrations were as described above, except that a single primer pair was used at a concentration of 1.5 μM for each primer, and only 0.5 U of DNA polymerase were used in each reaction. The PCR temperature profile was the same as in the first amplification step. Amplification products of the correct size for the two extinct bears were cloned using the TOPO TA cloning kit (Invitrogen), and a minimum of three clones were sequenced on an ABI3730 capillary sequencer (Applied Biosystems). For the modern samples, PCR products were either sequenced from both directions, or multiple clones were sequenced to ensure sequence accuracy. Primers for fragments that gave no product in the first amplification attempts were redesigned if the adjacent fragments showed substitutions in the primer site. The resulting primers were then used to amplify the remaining segments of the bear genomes. For the two extinct bear species, each position of the mtDNA was amplified at least twice from independent primary amplifications to ensure the authenticity of the sequence [13]. For the cave bear a nested primer design was chosen where the primers in the singleplex amplification are shifted inwards compared to the primers used in the multiplex step. This design ensures specificity of the singleplex PCR and reduces the risk of contamination of the multiplex PCR since only products from the singleplex reaction are amplified to high copy numbers [51]. All primer sequences used can be found in Additional File 1. For the sequenced modern bears and the cave bear primer sets EVEN and ODD are comprised of 20 primer pairs each. A single primer pair, EVEN21, spanning a repeat region within the D-loop, was excluded from both sets and only used in singleplex PCRs. For the Giant short faced-bear 81 primer pairs were designed in total and split into two sets; all amplification attempts spanning the repeat region within the D-loop for the American giant short-faced bear failed.

In Cambridge, amplifications were completed using the same PCR conditions as in Leipzig, but with a reduced number of primer pairs. Both water controls and an extraction control consisting of a mammoth DNA extract were negative for cave bear-specific products. Eighteen amplification products, originating from independent primary PCRs, were sequenced in both directions for 9 different fragments distributed throughout the whole mtDNA. A total of 3,520 bp were amplified and sequenced. The consensus sequences for all fragments were identical to the corresponding sequences produced in Leipzig.

For the American giant short-faced bear in total 395 bp of the mtDNA were replicated in two fragments at the Australian Centre for Ancient DNA. Details can be found in Additional File 1.

### Mitochondrial genome sequence alignment and annotation

The newly sequenced mtDNAs for the two extinct and five extant bear species, as well as the four publicly available genomes (three bears and a harbor seal) were aligned in MUSCLE 3.6 using the default parameters [52]. The D-loop was removed for all analyses, as it is too variable for interspecific comparisons and could partially not be determined from the American short-faced bear. We employed nine sequence data partitioning schemes in the following order: the transcription process; the three codon positions on each strand, the tRNAs on each strand and the rRNA genes. A few nucleotides were duplicated in the partitioned dataset because of the overlap of some loci, and a small number of non coding nucleotides were excluded. The annotation was completed using the program DOGMA [53] and modified manually to avoid overlap of tRNA and protein-coding genes.

### Phylogenetic analyses

The substitution model was selected using Akaike's Information Criterion on all models available in the baseml program of PAML 3.15 [54]. For both the partitioned and the unpartitioned datasets, the GTR+Γ [55-57] was found to be the best-fit model. This model was used in all subsequent analyses unless specified otherwise.

The phylogeny of the mtDNAs was reconstructed using a thorough maximum parsimony (MP) search that is implemented in TNT [58], with 500 random-addition sequences and a variety of tree space exploration techniques. We also employed maximum likelihood (ML) in RAxML 2.2.3 [59], as well as a Bayesian inference (BI) of phylogeny in MrBayes 3.1.2 [60]. The GTR+Γ_4 _substitution model was used for both ML and BI analyses. Phylogenetic support was provided with 1000 bootstrap pseudoreplicates in ML and 5,000 replicates of symmetric resampling in MP. MrBayes was run twice for 3 million generations with a burn-in of 2,500 steps. For details, see Additional File 1.

Incongruence between individual partitions was evaluated in an MP framework employing variations of Bremer support measures, as implemented in Automated Simultaneous Analysis of Phylogenies [61], as well as with the ILD test [62]. Agreement or disagreement between individual partitions at each node in the mtDNA tree was expressed through positive and negative hidden branch support (HBS) values, respectively [63]. See Additional File 1 for further details.

### Contrasting alternative phylogenetic hypotheses

We collected 10 alternative topologies on the phylogenetic relationships of bears from the available literature (see Additional File 1, Figure S2) and compared them in an ML framework using the approximately unbiased test (AU) [64] in CONSEL [65], along with a comparison of homoplasy indices and tree lengths.

### Estimation of divergence times

Dating of the divergence events within bears was done using a molecular clock approach and several fossil calibration points. The minimum for the divergence of bears and seals was set to 33.9 Ma, based on the fossil species *Parictis montanus *[66] and *Parictis parvus *[67] both dated to 38–33.9 Ma, and the first well-described members on the bear lineage. As a second calibration point, the minimum age for the oldest described *Ursus *fossils, *U. minimus *and *U. ruscinensi*s, at 4.2 Ma [47] was used, and the maximum for the youngest fossils from the genus *Ursavus*, *U. depereti *and *U. ehrenbergi*, which gave rise to the *Ursus *lineage [11], at 7.1 Ma [47].

The above mentioned calibration points were used as priors to obtain the posterior distribution of the estimated divergence times. Evolutionary rate constancy according to a molecular clock for all bear mtDNAs, including the harbor seal outgroup, was tested using a likelihood ratio test (LRT) in baseml [68]. The assumption of a molecular clock at the 1% level under a GTR+Γ model for the whole mtDNA alignment excluding the D-loop for the partitioned (-2δ*L *= 14.3, *p *= 0.112) and unpartitioned (-2δ*L *= 18.0, *p *= 0.036) dataset could not be rejected.

We estimated divergence times using two Bayesian approaches implemented in the programs mcmctree [54] and BEAST 1.4.4 [69]. Mcmctree was run using the HKY85+Γ_8 _substitution model [57,70], the most parameter-rich model available in this program. A total of 10^5 ^generations were sampled every 5 steps after discarding 10^4 ^initial steps as burn-in. The more parameter-rich model GTR+Γ_4 _was used in BEAST with the following priors: Yule speciation prior on the tree, siteModel.alpha (initial = 0.2, exponential prior with mean 1.0 and 95% CI of 0.05129–2.996), clock.rate (initial = 0.015, uniform prior of 0–10), root.height of ursine bear clade (uniform prior of 7.1–4.2 Ma based on the basal ursine bear radiation fossil data). Thirty million Markov chain Monte Carlo (MCMC) steps were sampled every 1,000 generations. Convergence was assessed in Tracer v1.3 [71] after excluding the first 5 million samples as burn-in. All effective sample size values exceeded 20,000, suggesting a sufficient run length. The strict clock was implemented in all divergence time estimations, as suggested by the LRT.

## Authors' contributions

JK, TU, AN, AWB, SJB, PHD, HS and MS were responsible for the experimental work. JK, ASM and SOK performed the sequence and evolutionary analyses. GR and PM obtained the ancient bear specimens and arranged the dating. LS contributed paleontological information on the bear fossil record. SJO provided extant bear DNA. AC, SP, JK, MSlatkin and MH conceived ideas for this project. JK, ASM, SOK and MH wrote the manuscript. All authors read and approved the final draft.

## Supplementary Material

Additional File 1 Supplementary materials. Click here for file
