# Supplementary material for: Mitochondrial genomes reveal an explosive radiation of extinct and extant bears near the Miocene-Pliocene boundary
Source: BMC Evol Biol. 2008 Jul 28;8:220. doi: 10.1186/1471-2148-8-220 (PMC2518930; doi:10.1186/1471-2148-8-220)
Supplement: Additional File 1 — Supplementary materials. [file 1471-2148-8-220-S1.doc]

**Supplementary Material**

**Ancient DNA sequence validation**

When comparing the sequences derived from a single primary amplification with those from another independent amplification, we found consistent differences at between one and twelve nucleotide positions, for 23 fragments from the cave bear and for one position in the American giant short-faced bear. In these cases, we either used the sequences of overlapping adjacent fragments or performed a third round of primary PCRs and used the majority consensus sequence to infer the correct sequence. All 82 consistent differences in the clone sequences for the cave bear and the American giant short-faced bear were found to be either C to T or G to A substitutions. In each case, a third independent product showed the consensus state to be C or G, respectively. To further ensure reproducibility of the sequence, 9 regions of the cave bear mtDNA were replicated in an independent laboratory in Cambridge. These regions were dispersed throughout the genome and represent a total of 3,520 bp.

No differences were observed in the consensus sequences through the overlapping regions between adjacent fragments for neither the modern nor the ancient mtDNAs. All sequences, except for the panda, showed highly similar base compositions (Table S1), an indication that none of the sequences was significantly affected by errors. The giant panda sequence differed slightly from the others by having a lower GC content. However, because this sequence was determined from modern DNA, it is likely to be accurate.

**Extraction and amplification procedures performed at the Australian Centre for Ancient DNA**

The American giant short-faced bear DNA extraction was performed in the Australian Centre for Ancient DNA from 250 mg of bone powder obtained from a calcaneum bone from Eldorado Creek, Canada, dated at 22,417 ± 452 years BP (CMN 37957). The extraction was done by first removing the exterior surface of the bone sample using a Dremel tool; subsequently 250 mg of the cleaned bone was powdered using a 9 mm tungsten ball bearing in a Mikro-Dismembrator (Sartaurius) at 3000 rpm in sterilized stainless steel canisters. The powder was then decalcified overnight in 10-20 ml of 0.5M EDTA (pH 8) on a rotary mixer at room temperature. The decalcified bone sediment was collected by centrifugation and digested in 3 ml of digestion mixture containing 100 mM Tris-HCl (pH 8), 100 mM NaCl, 0.5 mg/ml proteinase K, 10 mg/ml dithiothreitol (DTT), and 1% sodium dodecyl sulphate (SDS). Digestion was carried out overnight at 55°C on a rotary mixer. Following digestion, an equal volume (3 ml) of Tris-saturated phenol was added and mixed on a rotary mixer for 10 min at room temperature, followed by centrifugation at 1500 g for 5 min. The aqueous phase was transferred to a new tube, and the organic phase discarded. This process was repeated twice, once more with an equal volume of Tris-saturated phenol, and then once more with an equal volume of chloroform. The aqueous phase from the chloroform step was purified with sequential additions of DNA-free water to an Amicon Ultra-4 Centrifugal Filter Unit (Millipore), and concentrated to a final volume of 100 μl.

In Adelaide PCR amplifications were performed in 25 μl reactions with 1 μl of DNA extract, 1-1.25 U Platinum Taq DNA Polymerase High Fidelity and 1X buffer (Invitrogen), 2 mg/ml rabbit serum albumin (RSA; Sigma), 2 mM MgSO_4_, 250 μM of each dNTP, and 1 μM of each primer. PCR thermal cycling reactions consisted of 94 °C 1 min, followed by 50-55 cycles of 94 °C denaturation for 15 sec, annealing for 15-20 sec at 55 °C, and extension at 68 °C for 30 sec, followed by a final extension at 68 °C for 10 min. For the American giant short-faced bear in total 395 bp of the mtDNA were replicated in two fragments at the Australian Centre for Ancient DNA.

**Phylogenetic analyses**

A maximum parsimony search was carried out in TNT [1] employing a combination of sectorial searches, tree-fusing, and ratcheting (364,013, 250,248, and 4,680,248 rearrangements, respectively) with 500 random-addition sequences in order to thoroughly explore tree space[2, 3]. Nodal support was provided with 5,000 replicates of symmetric resampling[4]. Maximum likelihood phylogenetic inference was carried out in RAxML 2.2.3 [5] using a GTR+Γ_4_ model of substitution. Clade support was assessed with 1000 bootstrap replicates. Bayesian inference of phylogeny was undertaken in MrBayes 3.1.2 [6] with a GTR+Γ_4_ substitution model [7-10] and otherwise default parameters for MrBayes. The Metropolis-coupled Markov chain Monte Carlo was run twice for 3 million generations with a 1000-step thinning. Convergence was assessed graphically and 2,500 samples were removed as burn-in.

Alternative topologies were compared using Shimodaira’s approximately unbiased (AU) test [11]. Sitewise likelihood values for all 11 competing topologies were computed in PAUP[12] for both HKY+Γ_4_ and GTR+Γ_4_ substitution models (results did not differ between models). AU *p*-values were calculated in CONSEL [13] using the multiscale bootstrap method and 100,000 replicates. Low *p*-values (<0.05) indicate significantly worse topologies. Results are shown in Table S3. Competing topologies are shown in Figure S2.

For the estimation of the divergence times using mcmctree, we used Γ-distributed priors for kappa (κ), the transition transversion rate ratio, for alpha (α), the shape parameter of the Γ distribution of rates across sites, and rgene, the substitution rate. The Γ distribution parameters for κ were 0.001 and 0.0033, 1 and 5 for α, and 1 and 2 for rgene. We chose an uninformative prior for κ due to the lack of prior knowledge for this parameter; in addition to that we noticed that the posterior estimates were sensitive to the choice of κ priors. The fine-tuning parameters were set to 0.6, 0.5, 0.2, 0.2, and 0.2 so as to achieve intermediate acceptance proportions between 10% and 80% [14].

Phylogenetic incongruence among mitochondrial loci and partitions was quantified in terms of branch support (BS; also known as decay index or Bremer support) between data loci/partitions and for each node in a parsimony setting. First, the partitioned branch support (PBS) values were calculated for each partition and node with respect to the simultaneous analysis (also termed “total evidence” analysis) tree [15]. Then, the PBS values were summed to form the BS value for each node on the simultaneous analysis tree. Third, the locus/partition-specific BS values are calculated for each node on the simultaneous analysis tree then summed to reflect the agreement or disagreement of each partition with the combined dataset (SIBS; summed individual branch support). The difference between BS and SIBS is termed hidden branch support (HBS;[16]) and is an index that acquires positive and negative values when the individual partitions at each node on the simultaneous analysis tree agree and disagree, respectively. The incongruence length difference (ILD) test was used in pairwise locus comparisons [17].

All control files for the programs used can be made available upon request.

**Figure S1.** Geographical distribution of the bear species investigated. a) Worldwide distribution of modern bear species after Servheen [18]. b) Distribution of the two extinct bear species investigated, both from the late Pleistocene

a)

b)

**Figure S2. Competing hypotheses for the phylogeny of bears.** Hypothesis No. 9 is the one supported by our data. Hypothesis No. 6 is the competing hypothesis that was not rejected as a significantly worse topology by the AU test (Table S3).


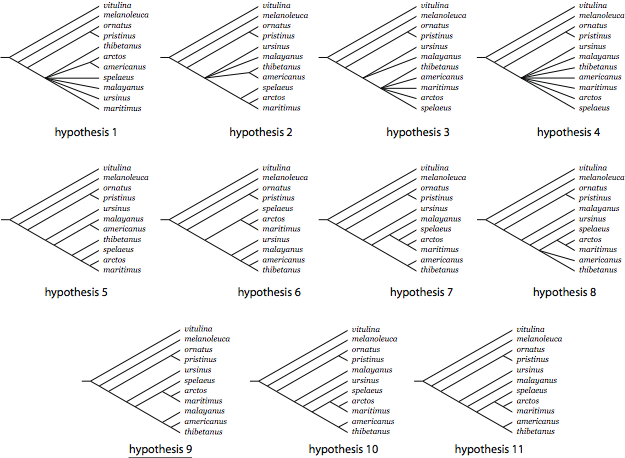


**Figure S3. Posterior 95% CI widths plotted against the posterior means of node ages for the unpartitioned dataset.** The adjusted correlation coefficient *R^2^* is 0.9997 (*p* < 2.2 ×10^-16^), indicating a saturation of the amount of data. A very similar graph, with *R^2^*= 0.9996 (*p* < 2.2 ×10^-16^), was obtained for the partitioned dataset (data not shown).


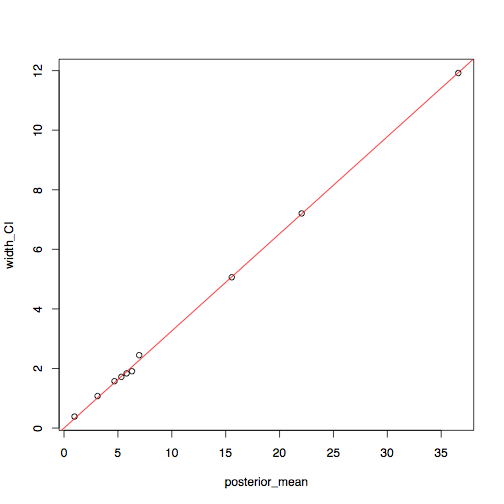


**Table S1. Overview of size and nucleotide content for each mtDNA from the bear species used in this study.** Nucleotide base (T, G, C, A) values indicate the frequency (%) of each nucleotide on the light strand of each mtDNA. *Gen Bank annotations for protein and t-RNA coding regions were modified to obtain concordant regions for all eleven mtDNAs. The annotated Gen Bank files can made be available on request.

Species name accession # mt size (bp) T G C A

Ursus americanus (NC_003426*) 16841 27.9 15.1 25.3 31.7

Ursus thibetanus (FM177759) 16893 27.8 15.1 25.6 31.6

Ursus arctos (NC_003427*) 17020 27.9 15.1 25.5 31.5

Ursus spelaeus (FM177760) 16780 27.9 14.9 25.4 31.8

Ailuropoda melanoleuca (FM177761) 16796 29.5 14.6 23.6 32.3

Ursus maritimus (NC_003428*) 17017 27.9 15.2 25.5 31.4

Ursus ursinus (FM177763) 16809 27.3 15.4 26.1 31.2

Termactos ornatus (FM177764) 16752 27.2 14.9 26.0 31.9

Ursus malayanus (FM177765) 16792 . 27.7 15.0 25.7 31.6

Arctodus simus (FM177762) 16361 (without repeat) 27.8 14.6 25.5 32.0

Harbor Seal (NC_001325*) 16826 27.4 14.3 25.3 33.0

**Table S2. Primer sequences used to amplify the five modern and two ancient bear mtDNAs.** Specificity to a certain bear species is indicated after the primer name. SP = spectacled bear, Pa = panda, AB = Asiatic black bear, Sloth = sloth bear, Face = American giant short-faced bear, out = outer primer pair for the first step Multiplex PCR used in the nested amplification protocol for the cave bear.

| Primer name | Forward primer 5’-3’ | Reverse primer 5’-3’ |
| --- | --- | --- |
| EVEN 1 | CAAGGAGCTTATGCACATTTA | CCAAACCTTTATGTTTATGGA |
| EVEN 1_Pa_Sloth | CCCCGTAACTTCAAGGAG |  |
| EVEN 1_SP | CAAGAAGCCTACTACGCGCATTTA |  |
| EVEN 2 | TCAGTAACACTAAAATAAGCTACGA | CCACTTGAGGAGGGTGAC |
| EVEN 2_AB | CCAATAACACTAAAATAGACTACGA |  |
| EVEN 2_SP | CGTCGATAGCACTAAAATAAACTAC |  |
| EVEN 2_Pa | CTACGAAAGTGACTTTAAAATTTCTG |  |
| EVEN 3 | GGGAATGATGAAAGATTACTT | CATACCTGTGTTGGATTAACAA |
| EVEN 4 | CAAAATAACCTCCGAGTGA | ATGTAGCCCAGTACTTTTCG |
| EVEN 4_Sloth | ATAAAAGAACCTCCGAGTGA | TTACGGAGTTGTATATAGCCTAA |
| EVEN 4_SP |  | ATGTAGCCTAGTACCTTCCG |
| EVEN 5 | CTATCCTTTGATCGGGATG | AAGTTGACAGTGTATAGTTCTGGT |
| EVEN 5_SP | TATTCTATTCTCTGGTCTGGATG | AAGTTGACGGTATATAGTTCTGGC |
| EVEN 5_Pa | GCCTTAACACTAGCTCTGAC | AACAATGTATAATTCTGGTATATAAGG |
| EVEN 6 | CCCTTCCCGTACTAATAAAA | CGGAGTTGAGTTTGGTTTAG |
| EVEN 6_Pa |  | TCGGAGTTGGGTTTGATTTAATC |
| EVEN 7 | AAAATGACAATTTGAAAGCA | GGCTGATAAAAGCACTAGACT |
| EVEN 7_SP | GAAATGACAATTCGAGACCACAA |  |
| EVEN 8 | CCTCCTCTATGGTAGAAGCA | GGGATAGCAATAATTATGGT |
| EVEN 8_SP |  | GTTGGGATTGCAATAATCATGGTT |
| EVEN 8_Pa | CTGATTACTACCTCCATCCTTTC | GAGTGGCTAGTCAGCTAAATACT |
| EVEN 9 | CATGAGCAAAAATTCACTTTA | AACACAATTATTAATGTATGGTCA |
| EVEN 9_SP |  | AATACAATCATTAATGTATGGTCGTG |
| EVEN 9_Pa | AACATTCTTCCCCCAGCACTT | CCTGTTTGAAAGGGATGCGC |
| EVEN 10 | CCGACACAAGAACTAAAGC | AAATAAAATGCTCGGAAATA |
| EVEN 10_Pa_SP |  | TCGTTGGGGTAATAAAAGAG |
| EVEN 11 | CTACCTCAAGGAACACCTTT | TTGAAATGTACTCTCTCGAATAA |
| EVEN 11_Pa | CTACCCCAAGGAACACCTCT | CATCATTGATATATGGTGAGTATGTT |
| EVEN 11_SP |  | GTGGTGGCCTTGAAAAGTA |
| EVEN 12 | ATCCGGAGTATCCATTACCT | TCAAACCCGCATTCATAG |
| EVEN 12_SP | AGCCCACCATAGCTTAATAG | GCGATTTCCAGGTCAAATAATAG |
| EVEN 12_Pa |  | AGAAGTGCTAGAATTAGGTTTATTAC |
| EVEN 13 | ACATAATTATCAAGTGCCTGTAG | TGGTGATGTATAATTTCTTTCG |
| EVEN 13_SP | AATGATTTCGACTCATTAGATT |  |
| EVEN 13_Pa | CAAGTGATTTCGACTCATTAGATTAT | TTGTGATATATAATTTTTTCCGGGTC |
| EVEN 14 | TTTCTGTGACTAGCGTGTATAA | GTTTGCAAGACAGAATAGTA |
| EVEN 14_SP | CATTCATGGTAAAAATACCC | GGTTCGTTCATAGTTGGAGT |
| EVEN 15 | AAAACGCCCTGATAGCTC | TTAGCTTTAGGGTTTGAATAGTT |
| EVEN 15_SP | ATCCTTCACACGAGAAAAC | TGTCTCTCAGTGTCAGTTCGAGA |
| EVEN 15_Pa | CCATCTTATACACGAGAAAATGC | AGTTATTCAATGTCAGTTTGAGA |
| EVEN 16 | TCTAGTGACCGCAAATAATCT | GCACAGGCTGCTGTAAATA |
| EVEN 16_SP | CTTAGTGACCGCAAACAACCT | GCACAGATTGCTGTGAATAGG |
| EVEN 16_Pa | CTTAGTGACCGCAAACAACCT | GCGCAGATTGCTGTAAACAAG |
| EVEN 17 | GACATCCGAAAAATAGGAG | CTTATTGATAAGCTCATTATTGG |
| EVEN 17_Pa | CAAGATATTCGAAAGATAGGAG | AGTAGGAGATGGATTTTGGT |
| EVEN 18 | CATGGCTTCCTCACTAAAAA | AGATTCCATGTGGGAATAAT |
| EVEN 18_Pa | ACCTACAATAAATGCACCTAGTAC | GATTCCATGTAAGAATGATGATAACA |
| EVEN 19 | TGCCGAGACGTTCACTAC | AGAAGTAGGGCGCCTAGG |
| EVEN 19_SP | GGTCTGTACTACGGCTCATA | ATTAAGGCGAAGAGTAGGGC |
| EVEN 19_Pa | TTGTCGAGACGTCAACTATGG | GAGGAATAGGGCACCTAGG |
| EVEN 20 | ATTGGAGGACAACCAGTAGA | GTACTCGCAAGGATTGCT |
| EVEN 21 | AAGGGGTTACTCAGTCAATG | TCTTGTTTTTGGGGTTTG |
| EVEN 21_SP | ATAAGGTGTTATTCAGTCAATGGTTA |  |
| EVEN 21_Pa | ATAAGGTGCAATTCAGTCAATGG |  |
| Primer name | Forward primer 5’-3’ | Reverse primer 5’-3’ |
| ODD 1 | CTGAAAATGCCTAGACGAGT | GATCTTAGCTGTCGTGTGG |
| ODD 1_Pa |  | GGGCCCTAGCTATCGTGTAA |
| ODD 2 | GCTTGATTGAATAAGGCAATG | AATCTTTGCTGTTTGTTACTTTA |
| ODD 2_Pa |  | AGAAGGGGCAATCTTTGCTGTT |
| ODD 2_SP |  | GGTAAAAGAAGTAATCTTTGCTGTTT |
| ODD 3 | ATAAAACACCTACCAAACCA | TGGATCAATAAGTAAGTGAGCA |
| ODD 3_Pa | CCACTGATAGTTAACAACAACATAG | GGATCAATAAGTGATTAAGCATTTTG |
| ODD 3_SP | CCACTGATAGTTAACAACGATATAG |  |
| ODD 4 | CCGTAGCCTTCTTAACACTA | GCGTATTTTGAGTTTGAAGC |
| ODD 4_Pa | GCTGTAGCCTTTCTGACTCT |  |
| ODD 5 | CTTCGGAGCATTTCACAGCC | GATGTGATAATGATAAGAATGGGTG |
| ODD 5_SP | TCCTATTCTTTGGAGCATTCCAT | TGAGTCAATGGGAGCTAAAT |
| ODD 5_Pa | CTTCGGAGCATTTCACAGCC | ATGTAATGGTAATAAGGATGGCTG |
| ODD 6 | GTATTAGTAGGAGGCTGAGGA | TGGTTGATGCTACGATTAGA |
| ODD 6_SP |  | ATAGTTGATGCTACAATCAGAGG |
| ODD 6_Pa |  | TAGTTGATGTCACGATTAGAGGG |
| ODD 7 | CAATTCAATATGATTATTCACCA | GTTCATCCAGTCCCTGCA |
| ODD 8 | CACGAGCTTACTTCACTTCA | CTGAGGAAAGAATGTCATATTAAC |
| ODD 8_Pa | CGAGCCTATTTTACCTCGGC | GAATATCGTCGAGGCATACC |
| ODD 9 | TCAGGGTTAAATTATAGGTGAAA | TCTAATAACCGCAATTCTCCA |
| ODD 9_Pa |  | ACTACTCGATTATCTACCTCTAG |
| ODD 10 | TGAGAAGAAAAATGAACGAA | CAAGTGGCCTGCAGTAATA |
| ODD 10_SP | ACCTTGAGAAAAGAAATGAACGAAAAT | AGTAAGTGACCTGCAGTGATG |
| ODD 11 | TAATAGGAGCCCTTTCAGC | GGCCTGGAGTATGTGCTTG |
| ODD 11_Sun | CCTTTCAGCCCTTCTAATAA | ACGCCTAGAGAGATAGTAATAAAT |
| ODD 11_SP | TAACGGGAGCCCTCTCAG | AGCCTGGAGTACATGATTGC |
| ODD 11_Pa | CGGTATACTTACTAATACTAGGC |  |
| ODD 12 | GCCTTCTGACTACCACAGTTA | GGGATACAATGAAGGCTAAGA |
| ODD 12_SP | CGCATTCTGGTTACCACAATTAT | GGGATACAATAAAAGCCAAGAAG |
| ODD 12_Pa | TCAAGCTCTGGTATAATCCAGAA | TTAAGGATATAGTAAAGGCTAGAAAG |
| ODD 13 | CAATTTCACCTGTCAAAGGA | CCTCTACGTGTGCTTTTGGT |
| ODD 14 | TTAATCCAAACCCCATGA | ATTTTGGGATTAAGGGATAGTAGC |
| ODD 14_AB | GAGCAACAGCCCTAATAGTT | TGGGGTTGAGAGATAGAAGT |
| ODD 14_SP_Pa | TAGCCCTAGTCATTGTAGCA | GCGTACTTTTTCGGTAAATAA |
| ODD 15 | AGCGCTTCAACACACAAA | GGAAAGATATGATCCCTACTCC |
| ODD 15_SP | TTATCCTAACCAGTACTTCAACATAT |  |
| ODD 15_Pa | AATCCATTGGTCTTAGGAACCAA | GAGAAATGATATGATTCCTACTCC |
| ODD 16 | GCAGGGGTATTTCTTCTAATC | AGAATCCTGTTAGGAAAGGTAT |
| ODD 16_SP | GCAGGGGTATTCCTCCTAATT | AGAACCCCGTAAGGAAAGGCAT |
| ODD 16_Pa |  | GTAAAATCCTGTTAAGAAGGGCAT |
| ODD 17 | CACAAAATCCATTCAAATTC | ACTGAGCAGTATCCTGAGGT |
| ODD 17_SP | CCACTAAGCCCATTCAAATTTTC | GCTACTGAGCAGTATCCTGAA |
| ODD 17_Pa | CCCTTTAAGCACGTTCAAATTTTC |  |
| ODD 18 | GGAGAAGGCTTAGAAGAAAA | CCGATGTTTCATGTTTCTG |
| ODD 18_Sun | CCCATCACAAAGATAGTACT |  |
| ODD 19 | AATCCCATTCCATCCATAC | GGAGGCTAGTTGTCCGATA |
| ODD 19_SP | AATCCCATTTCACCCATACTATAC |  |
| ODD 19_Pa | CTGACCCAGACAAAATCCCAT | GAATAGAGGCCAATTGCCCAATA |
| ODD 20 | AAGCATGTATTTCACTTAGTCC |  |
| ODD 20_SP |  | TCTCTACGTGTCGTATGTCC |
| ODD 20_Pa |  | TGATCTATAGTGTTATGTCCTGTG |
| ODD 20_Sloth_AB |  | GGTATTTACATACTATATGTCCCGTA |
| ODD 20_Sun | TAGTCCGGGAGCTTAATCA | GTCGTTCGTTCGATTTAGTG |
|  |  |  |
| Primer name | Forward primer 5’-3’ | Reverse primer 5’-3’ |
| EVEN1_Face | GGATACAGCAGTGATAAAAATTA | GCTAGTAGTTCTCTGGCGAAT |
| EVEN2_Face | ATCTTCAGCAAACCCTTAAA | AAATCCACCTTTAGTTTTTAGTTT |
| EVEN3_Face | GCGTACTGGAAAGTGTGCT | AAGTAATCTTTCATCGTTCCC |
| EVEN4_Face | GGTAGAGGTGAAAAGCCTAA | TATTTACTCTCTAAGTAAGGCTGTA |
| EVEN5_Face | CTCCTAACATGATAACTGGGCCAA | CCGTTTAACTAATGTCACC |
| EVEN6_Face | ACTTGTATGAACGGCCACA | AGTGAGCATTTCGACTAGT |
| EVEN7_Face | ATCCCAATGGTGCAGCAG | CTGCCACCCTAACAAAGC |
| EVEN8_Face | CCGTAGCCTTCCTAACACTA | ATCCGGATCAGAGAATAGA |
| EVEN9_Face | ATGAAGTCACATTAGCCATT | GTCGTGAGGGCATTTATTAT |
| EVEN10_Face | CTGCCTATTATTACAGCAAG | ATGAATCAGTGGGAGCTAAA |
| EVEN11_Face | ATCCCCATTCTAATAAAAAA | ATAGATAAAGGTGCGATTT |
| EVEN12_Face | ATCATAATAACTCTAGGGACATTT | AATAAAGGTTTAGTAGGGCTGT |
| EVEN13_Face | CCTCTAATCGTAGCATCAAC | CAGTTGTTTGGGGTTTTTA |
| EVEN14_Face | ACCACAGGGCTTGGCAAA | GATTTGATCATCCCCTAAC |
| EVEN15_Face | ATAGTAGAAGCAGGYGCA | AGTATTGAGGTTTCGGTCTGT |
| EVEN16_Face | CATATTGTCACATACTACTCAG | ATTGGCCAAGACAATTCCTGTGA |
| EVEN17_Face | AATACATGAGCAAAAATTCAC | CCACCTCTCGTTTTGATG |
| EVEN18_Face | TCTCTCAATAAAGAGATATTAGTAAAA | ACTTCTTGTGCGTCTATTGT |
| EVEN19_Face | TGATATTGAAGTTACGAGTATACAG | ACAAATTTCTGAGCATTGG |
| EVEN20_Face | CACAGCATTAACCTTTTAAGTTAA | ATCGTTGGGGTAGTAAAAGA |
| EVEN21_Face | AGGGACAAACCTGAGCACTG | GGTGACCCGCAGTGATATT |
| EVEN22_Face | CCTAGTACTACTCACTATTCTTG | GAGTGTGGTGGCCTTGAA |
| EVEN23_Face | CTCCCGAACTAGGAGCAT | AACCCAGTTGCTACGAAGAA |
| EVEN24_Face | GATGAGGATCCTGCCTCT | CTCGTAATGATCAGGGAAG |
| EVEN25_Face | ATCATAATTATCAAGTGTCCAT | GATAATTTTTAGCATTGTAGGAGG |
| EVEN26_Face | AATATCAAAACCCAATATAA | GGTGGCCTCAAATAGGATR |
| EVEN27_Face | CGGGTCTTTACTTTCTWTT | CCTCCTAGTTTTAGAAGTACG |
| EVEN28_Face | AATCCCTAATTGCCTACTCCT | GGAGTCATCAGGCTGCTATG |
| EVEN29_Face | ATTAATTACCACACAACGAGG | CCTAAGACCAATGGATTACTY |
| EVEN30_Face | ACCCACAGTACGTAAAGACCACA | GTTTGCGGTTACTAAGGTTATC |
| EVEN31_Face | GGCTATAGCCTGATTTCTACT | ATTAAGAGAAAAACTCCTGCC |
| EVEN32_Face | GATTAATAATCGTGACAATTGGA | AAGGGTTGTTAGTAGGGCCCA |
| EVEN33_Face | CCTCGCAGGATATTTAATCT | GATTTTATTTGAAAGTAGGAGA |
| EVEN34_Face | CATGGCCTCCTCACTAAAA | GTTAGTGGGGGTGTTGGG |
| EVEN35_Face | AATGACCAACATCCGAAA | TGAGAATAGGTATGAGCCATAG |
| EVEN36_Face | AACTATAGCCACAGCATTC | GTTGGATGGAATTCCGGAG |
| EVEN37_Face | ATTTGCCTACGCTATCCTAC | CAAGGTAATTGCTATACTACAAAGA |
| EVEN38_Face | CCCTAAGACTCAAGGAAGAA | GGCCAAGCACAATATGTAC |
| EVEN39_Face | CATCTGGTTCTTACCTCAGG | GACTGAGTAACCCCTTATGG |
| EVEN40_Face | GATATTAACTTAGCTTAATCAAACC | GCGATAATAATGGAAAAATCAA |
| EVEN41_Face | CTGAAAATGTCTAGATGAGT | AGTCGAACTTTCGTTCATGG |
| Primer name | Forward primer 5’-3’ | Reverse primer 5’-3’ |
| ODD1_Face | CACTATGCTTAGCCTTAAACA | GGTTACACCTTGACCTAACTTT |
| ODD2_Face | TGGGCTACATTTTCTATTCA | ATGCTTTGTTTAAGCTACACTT |
| ODD3_Face | GAGCTATAGAGAAAGTACCGC | AATTCTATTCTGGGCAACC |
| ODD4_Face | AAATATATTCTAAAAAGGTACAGCTT | CATATTAGCATTGTTGCTTCTA |
| ODD5_Face | CTCCAGCATTTCTAGTATTG | CTTTTGGGTTAATTAATTAAAGCT |
| ODD6_Face | ATAAAATAACCTCCGAGTGAT | TTTAAATAGATAGAAACCGACCT |
| ODD7_Face | CGCCTTAAGACCAATAGATG | GTAGGGTCCTACAATGTTTG |
| ODD8_Face | TTTATTATAGCCCCTATTCTAGC | AATGAGCCATTTATTAGTAGGA |
| ODD9_Face | GCCCTATTTTTCCTAGCAG | AAGTAATTCTTTTGTCAGACATATT |
| ODD10_Face | CCCTTCCCGTACTAATAAAA | TGTGTGAGGAAATATTTTGTAGA |
| ODD11_Face | TCCTCAGGAATGATTCTACTAA | GGACAGTGATAGTGTTGTTGT |
| ODD12_Face | GCTTTATTCCCAAATGAATA | TGGAGTTAGTGGGAGGAGTA |
| ODD13_Face | TATCCCACAAAATTTTAGTTAAC | CATAGGTAAAATGGCTGATAA |
| ODD14_Face | ACTGCCCTCAGCCTTCTA | GATGCTCCTGCATGGGC |
| ODD15_Face | ACTTCTTCTTTTATCTCTACCAGT | AAATCCTAAGAATCCAATGG |
| ODD16_Face | ATCCCCCGCTATAATATGAG | GTATCGTCGAGGTATTCC |
| ODD17_Face | CAGTATCTTCTATAGGCTCATTCA | CCATAAAGATATAAAGGATTTTCA |
| ODD18_Face | TCCTAATTAGCTCCTTAGTTCTC | CCAGGCTTTAGTTCTTGTGT |
| ODD19_Face | CCTAGGGCTAAAAACTGATG | GTTGTGGCATCTTCATTAAG |
| ODD20_Face | ATACCTTGAGAAAAGAAATGAAC | GCCTAGAAGGTTGGTTGAGCC |
| ODD21_Face | CTACCTCAAGGAACACCTTT | AAGTGTTATCGTGTAAGTATAGGC |
| ODD22_Face | CTAGGCCTTACAACTAATATAC | GACGCTAGGAGTACTGAGGT |
| ODD23_Face | CAGAATATTACGAAGCATCTTT | ATTGATTGGAAGTCAATTGTAC |
| ODD24_Face | CCTGGAAATCGCACTACTYCT | GGATACAATAAAAGCCAAGAA |
| ODD25_Face | ATTTGTCATGCTAACAGTAACAG | GAGAAGTTCAGGCTGTTATCGC |
| ODD26_Face | AGTCAATCCCACCTGTCAAA | ATCATGAGAAAGTTTAAGGAGCC |
| ODD27_Face | CCAAAAGCACACGTAGAAG | AATACTGCTACAATGACTAGGG |
| ODD28_Face | CCATAGCCGAACTATAATCC | GCTATCAGGGCGTTTTCT |
| ODD29_Face | ATTTACCGAAAAAGTACGCAAG | GACCGTTTCTTGTCCTGAG |
| ODD30_Face | GCCTGTTCGTCACATGAT | GATTTGTTGGAAGTCTCATG |
| ODD31_Face | GCCCTACTCCACTCTAGTACA | GCGTGTGTGCAGATATGA |
| ODD32_Face | AGGATTCTATTCTAAAGACCTGATC | TAGGGTATGGTTATTTGTGG |
| ODD33_Face | CCTGACTGGAAAATGTATTGCCA | ATTTATGATACGGGAGACTC |
| ODD34_Face | CCCGAAACTTAGTACGATACC | ATGAGTTATTGATAATTTTGGC |
| ODD35_Face | TGCCTATTCATGCACGTA | GTTGCCTTATCTACGGAAAA |
| ODD36_Face | CACCTACTATTTCTACACGAA | ACTAGGGCTAGTACTCCTCCTAGT |
| ODD37_Face | TCGCTGGAATTATTGAAAA | AAATAGAACATCAGCTTTGG |
| ODD38_Face | GTGCCCCATGCATATAAG | CGAGATGTCCCATTTGAA |
| ODD39_Face | Repeat region no amplification attempted |  |
| ODD40_Face | CTAAACGCACGTGCAAACAT | GGCCAGGACCAAACCTTTRT |
| Primer name | Forward primer 5’-3’ | Reverse primer 5’-3’ |
| EVEN 1_out | CCGTAACTTCAAGGAGCTT | GGCTAGGACCAAACCTTTAT |
| EVEN 2_out | TAATCCAGCTGTAAAAAGCTATC | CTTGTGCCACTTGAGGAG |
| EVEN 3_out | CAAGGGAATGATGAAAGATT | GCATACCTGTGTTGGATTAAC |
| EVEN 4_out | ACAAAATAACCTCCGAGTGA | CGAAGTTGTATATAGCCCAGT |
| EVEN 5_out | GCTTAGCTGTATATTCTATCCTTTG | AAGTTAACGGTGTATAGTTCTGGT |
| EVEN 6_out | CCCTTCCCGTACTAATAAAA | TAGGATTTTTCGGAGTTGAG |
| EVEN 7_out | GCAAACAATACAAAAATAAAATG | GAACATAGGTAAAATGGCTGA |
| EVEN 8_out | CCTCCTCTATGGTAGAAGCA | CAGCTAAATACTTTGACTCCTG |
| EVEN 9_out | ACATGAGCAAAAATTCACTTTA | GAGCTAATTAGGAACACAATTATTA |
| EVEN 10_out | CCGACACAAGAACTAAAGC | AGGAAATAAAATGCTCGGAAA |
| EVEN 11_out | CTTGGCTCACTTTCTACCTC | TTGAAATGTACTCTCTCGAATAA |
| EVEN 12_out | CTTCAGTACTTCTTGCATCC | TCAAACCCGCATTCATAG |
| EVEN 13_out | TAACATAATTATCAAGTGCCTGT | TGGTGATGTATAATTTCTTTCG |
| EVEN 14_out | TTTCTATGACTGGCATGTATAA | AGTTGGAGTTTGCAAGACAG |
| EVEN 15_out | GAGAAAACGCCCTGATAG | TTAGCTTTAGGGTTTGAATAGTT |
| EVEN 16_out | TCTAGTGACCGCAAATAATCT | AGAGCACAGGCTGCTGTA |
| EVEN 17_out | ATGAACAAGACATCCGAAA | CTTATTGATAAGCTCATTATTGG |
| EVEN 18_out | CATGGCTTCCTCACTAAAAA | TCATGGTTAGATTCCATGTG |
| EVEN 19_out | TCAGTCACCCACATTTGC | AGAAGTAGGGCGCCTAGG |
| EVEN 20_out | ATTGGAGGACAACCAGTAGA | GGTACACGTACTCGCAAGG |
| EVEN 21_out | CCGTAACTTCAAGGAGCTT | GGCTAGGACCAAACCTTTAT |
| Primer name | Forward primer 5’-3’ | Reverse primer 5’-3’ |
| ODD 1_out | AGCAAGGCACTGAAAATG | ATCCCAGTTTGGATCTTAGC |
| ODD 2_out | CAAGAATAGAGAGCTTGATTGA | GGTAGGAGGGGTAATCTTTG |
| ODD 3_out | ATCCAACAATAAAACACCTACC | TAAATAGGATTGCGCTGTTA |
| ODD 4_out | CTCGCCGTAGCCTTCTTA | CTCCAATCAGAGCGTATTTT |
| ODD 5_out | TCCTGTTCTTCGGAGCATTT | AGGATAATGGTGGATGTGAT |
| ODD 6_out | AGCCGCTACATCCGTATT | GGGAGGAGTATGGTTGATG |
| ODD 7_out | GAATTTGCAATTCAATATGATTA | GAGGATAGACAGTTCATCCAGT |
| ODD 8_out | ACGTTGACACACGAGCTTA | ACAGGCCTAGAAAATGCTGA |
| ODD 9_out | ACATAACTTTGTCAGGGTTAAA | GTCCACTTCTAATAACCGCAAT |
| ODD 10_out | CCCAACGATAATGGGGAT | AAATAGGCTGATTGTTTCGA |
| ODD 11_out | CCCATGACCACTAATAGGA | TGAATAGGGCCTGGAGTATG |
| ODD 12_out | TCGTTCTTATTGCCTTCTGA | GGGATACAATGAAGGCTAAGA |
| ODD 13_out | CGAGTCAATTTCACCTGTCA | ATAGGTGCCTCTACGTGTGC |
| ODD 14_out | TAATAATCGCCCACGGACTA | ATTTTGGGATTAAGGGATAGTAGC |
| ODD 15_out | TCCTCACCAGCGCTTCAA | CCCGATAAGGAGGAAAGATA |
| ODD 16_out | CAATAGTAGTAGCAGGGGTATTT | AGTAGAATCCTGTTAGGAAAGG |
| ODD 17_out | TCTCAAACTTGACTATCCACAA | ATTTATGATACGGGGGATTC |
| ODD 18_out | CAAAACCCATCACAAAAACG | CCGTCCTACGTGCATAAATA |
| ODD 19_out | AAAATCCCATTCCATCCA | TAAAGTAGAGAATGGAGGCTAGT |
| ODD 20_out | CACTTAGTCCGGGAGCTT | CGTCGTTCGTTCGATTTA |

**Table S3. Comparison of competing topological hypotheses for the phylogeny of bears.** Topologies are sorted by AU test *p*-value.

| **Hypothesis** | **Log-likelihood**  **(GTR+Γ_4_ model)** | **AU test**  ***p*-value** | **Tree length** | **Homoplasy index** |
| --- | --- | --- | --- | --- |
| 9 | -62752.2225 | 0.908 (best) | 10092 | 0.389 |
| 6 | -61652.0915 | 0.147 | 10157 | 0.393 |
| 7 | -62664.4542 | 0.011 | 10115 | 0.390 |
| 11 | -62752.2225 | 0.011 | 10103 | 0.389 |
| 10 | -61659.3074 | 0.007 | 10124 | 0.391 |
| 2 | -61628.5789 | 0.003 | 10303 | 0.401 |
| 5 | -61646.0214 | 0.001 | 10151 | 0.392 |
| 8 | -61671.7978 | 0.001 | 10243 | 0.398 |
| 3 | -61624.9899 | 10^-8^ | 11177 | 0.448 |
| 4 | -61650.2677 | 6×10^-8^ | 11334 | 0.456 |
| 1 | -61646.0224 | 6×10^-8^ | 11266 | 0.453 |

**References**

1. Goloboff P, Farris J, Nixon K: **TNT, a free program for phylogenetic analysis.** *Cladistics* 2008, **24**:1-13.

2. Goloboff PA: **Analyzing large data sets in reasonable times: solutions for composite optima.** *Cladistics* 1999, **15**:415-428.

3. Nixon KC: **The parsimony ratchet, a new method for rapid parsimony analysis.** *Cladistics* 1999, **15**:407-414.

4. Goloboff PA, Farris JS, Kallersjö M, Oxelman B, Ramírez MJ: **Improvements to resampling measures of group support.** *Cladistics* 2003, **19**:324-332.

5. Stamatakis A: **RAxML-VI-HPC: maximum likelihood-based phylogenetic analyses with thousands of taxa and mixed models**. *Bioinformatics* 2006, **22**:2688-2690.

6. Ronquist F, Huelsenbeck JP: **MRBAYES 3: Bayesian phylogenetic inference under mixed models.** *Bioinformatics* 2003, **19**:1572-1574.

7. Lanave C, Preparata G, Saccone C, Serio G: **A new method for calculating evolutionary substitution rates**. *J Mol Evol* 1984, **20**:86-93.

8. Rodriguez F, Oliver JL, Marin A, Medina JR: **The general stochastic model of nucleotide substitution**. *Journal of theoretical biology* 1990, **142**:485-501.

9. Yang Z: **Maximum-likelihood estimation of phylogeny from DNA sequences when substitution rates differ over sites**. *Mol Biol Evol* 1993, **10**:1396-1401.

10. Yang Z: **Maximum likelihood phylogenetic estimation from DNA sequences with variable rates over sites: approximate methods**. *J Mol Evol* 1994, **39**:306-314.

11. Shimodaira H: **An approximately unbiased test of phylogenetic tree selection**. *Syst Biol* 2002, **51**:492-508.

12. Swofford DL: **PAUP*. Phylogenetic Analysis Using Parsimony (*and Other Methods)**. Version 4b10. Sunderland: Sinauer Associates; 2002.

13. Shimodaira H, Hasegawa M: **CONSEL: for assessing the confidence of phylogenetic tree selection**. *Bioinformatics* 2001, **17**:1246-1247.

14. Yang Z, Rannala B: **Bayesian estimation of species divergence times under a molecular clock using multiple fossil calibrations with soft bounds**. *Mol Biol Evol* 2006, **23**:212-226.

15. Baker RH, DeSalle R: **Multiple sources of character information and the phylogeny of Hawaiian drosophilids**. *Syst Biol* 1997, **46**:654-673.

16. Gatesy J, O'Grady P, Baker RH: **Corroboration among data sets in simultaneous analysis: hidden support for phylogenetic relationships among higher level artiodactyl taxa**. *Cladistics* 1999, **15**:271-313.

17. Farris JS, Källersjö M, Kluge AG, Bult C: **Testing significance of incongruence**. *Cladistics* 1994, **10**:315-319.

18. Servheen C: **The status and conservation of the bears of the world**. In: *Proceedings of the International Conference on Bear Research and Management. Monograph Series No. 2.* Victoria: Mock and Associates Inc.; 1990:32.
